# Prospect of Stem Cells in Bone Tissue Engineering: A Review

**DOI:** 10.1155/2016/6180487

**Published:** 2016-01-06

**Authors:** Azizeh-Mitra Yousefi, Paul F. James, Rosa Akbarzadeh, Aswati Subramanian, Conor Flavin, Hassane Oudadesse

**Affiliations:** ^1^Department of Chemical, Paper and Biomedical Engineering, Miami University, Oxford, OH 45056, USA; ^2^Department of Biology, Miami University, Oxford, OH 45056, USA; ^3^Sciences Chimiques, University of Rennes 1, UMR CNRS 6226, 35042 Rennes, France

## Abstract

Mesenchymal stem cells (MSCs) have been the subject of many studies in recent years, ranging from basic science that looks into MSCs properties to studies that aim for developing bioengineered tissues and organs. Adult bone marrow-derived mesenchymal stem cells (BM-MSCs) have been the focus of most studies due to the inherent potential of these cells to differentiate into various cell types. Although, the discovery of induced pluripotent stem cells (iPSCs) represents a paradigm shift in our understanding of cellular differentiation. These cells are another attractive stem cell source because of their ability to be reprogramed, allowing the generation of multiple cell types from a single cell. This paper briefly covers various types of stem cell sources that have been used for tissue engineering applications, with a focus on bone regeneration. Then, an overview of some recent studies making use of MSC-seeded 3D scaffold systems for bone tissue engineering has been presented. The emphasis has been placed on the reported scaffold properties that tend to improve MSCs adhesion, proliferation, and osteogenic differentiation outcomes.

## 1. Introduction

Every year, more than 1 million surgical procedures involving the partial excision of bone, bone grafting, and fracture repair are performed in the USA, at an estimated cost of more than $5 billion [1–3]. A substantial percentage is for the elderly, the number of which is expected to double in the next 25 years [4]. Worldwide, fractures due to osteoporosis affect approximately one woman in three and one man in five over the age of 50 years and are a major cause of suffering and disability in the elderly population [5]. The repair rate of a bone defect is dependent on the wound size. When the defect size is greater than the healing capacity of osteogenic tissues, the fibrous connective tissue becomes dominant in the bone defect [2, 6]. Well-established clinical approaches are restricted to autograft and allograft transplantation. However, they are limited in availability and associated with postoperative complications [7, 8].

As an alternative, tissue engineering applies the knowledge of bioengineering, biology, cell transplantation, and materials science to construct biological substitutes that can restore and maintain normal function in injured and diseased bone [9, 10]. The tissue engineering approach often involves the use of mesenchymal stem cells (MSCs) that are seeded into 3D scaffolds and induced to generate new bone by osteoinductive cues [11]. A fundamental requirement for tissue-engineered bone grafts is the ability to integrate with the host tissues, while providing the capacity for load-bearing and remodeling [12]. The size of scaffold-tissue constructs that can be cultured is limited due to high metabolic activity of bone cells [11]. This poses an additional challenge in terms of providing an efficient transport of oxygen, nutrients, and metabolic wastes. Therefore, 3D scaffolds are designed to accommodate these mass transport requirements while offering a load-bearing matrix during the bone healing process [13]. Scaffold composition and surface properties also play a major role in MSCs proliferation and differentiation. Since bone is largely composed of hydroxyapatite (HA) [14], incorporating HA into scaffold formulation can enhance osteoconductivity [15, 16]. Nanostructured HA (nHA) has a higher surface area, and consequently higher reactivity [17], and can enhance MSCs adhesion, proliferation, alkaline phosphatase activity, calcium deposition, and osteogenic gene expression [18–20].

This paper briefly covers various types of stem cell sources that have been described in the scientific literature for use in tissue engineering applications. Then, an overview of some recent studies making use of MSC-seeded 3D scaffold systems for bone tissue engineering has been presented, while placing the emphasis on the recommendations made in these studies to further improve cell adhesion, proliferation, and osteogenic differentiation outcomes. The majority of these studies have focused on bone marrow-derived mesenchymal stem cells (BM-MSCs) due to their high osteogenic potential [21–27]. The prospect of MSCs for bone tissue engineering has been summarized in the concluding section of this paper.

## 2. Stem Cell Sources for Bone Tissue Engineering

Bone tissue engineering requires a reliable stem cell source, in addition to appropriate 3D scaffolds and growth factors. Control over the differentiation of MSCs makes them attractive cell sources for bone tissue engineering. Adult stem cells, induced pluripotent stem cells (iPSCs), embryonic stem cells (ESCs), and umbilical cord blood mesenchymal stem cells (CB-MSCs) are among the candidates for bone tissue engineering applications [28–30]. In addition, adipose-derived stromal vascular fraction (SVF) has been reported to be an effective and abundant source for vascularization strategies, where regenerating vascularized bone tissues is desired [22].  Table 1 lists some of the advantages and disadvantages associated with these stem cell sources [22, 28, 29]. The following section further elaborates on the potential of these cell sources for bone repair and regeneration.

**Figure 1:**
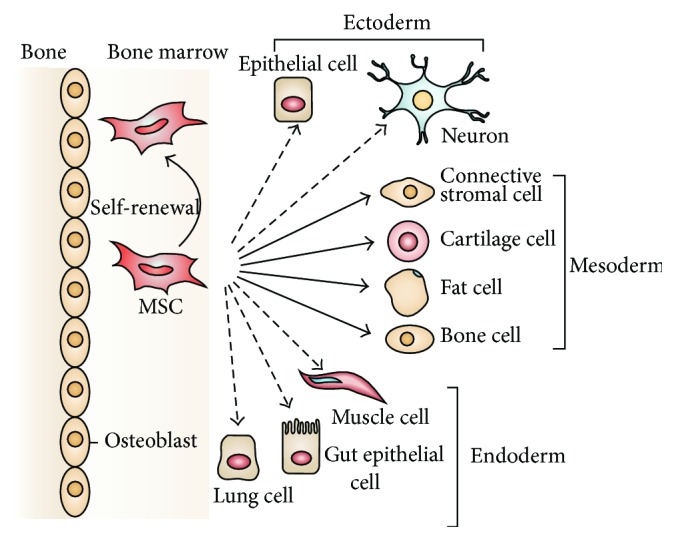
The ability of MSCs in the bone marrow cavity to self-renew (curved arrow) and to differentiate (straight, solid arrows) towards the mesodermal lineage (including bone cell). The reported ability to transdifferentiate into cells of other lineages (ectoderm and endoderm) is shown by dashed arrows, as transdifferentiation is controversial* in vivo*. Reprinted by permission from Macmillan Publishers Ltd.,* Nature Reviews Immunology*, Uccelli et al. [32], copyright © 2008.

A high volume of research in bone tissue engineering has been devoted to adult stem cells, which can be isolated from tissues such as a bone marrow or adipose tissue. In particular, bone marrow-derived mesenchymal stem cells (BM-MSCs) are attractive candidates due to their high osteogenic capacity [22]. As Figure 1 shows, MSCs in the bone marrow cavity can differentiate into cartilage, fat, and bone cells (mesoderm) and into several other cell types. Although stromal cells with similar characteristics can be isolated from almost any connective tissue [31], MSCs have mainly been characterized after isolation from the bone marrow [32]. However, relatively low abundance of BM-MSCs necessitates extensive* in vitro* expansion, which diminishes the posttranslational survival and immunomodulatory properties of BM-MSCs while posing regulatory and logistic challenges [22]. In addition, the donor and patient age is a critical factor that must be accounted for in laboratory and clinical evaluations [33].

Adipose-derived stem cells (ASCs) are capable of differentiating into various cell types and possess similar osteogenic characteristics as BM-MSCs [34]. Human adipose tissue is abundant and can be easily obtained under local anesthesia with minimal patient discomfort, since a liposuction procedure is less invasive than bone marrow aspiration [28]. In addition, 1 g of adipose tissue can yield around 5 × 10^3^ stem cells, making it 500-fold greater than the number of MSCs in 1 g of bone marrow [28, 35]. Clinical applications of ASCs are in practice today and show a great promise for future research. In a study, a 7-year-old female with posttraumatic calvarial defects was treated with autologous ASCs, fibrin glue, and a biodegradable scaffold. Postoperative new bone formation as well as relatively complete calvarial continuity was reported based on computed tomography analysis [28, 36].

Embryonic stem cells (ESCs) have a strong multilineage differentiation capability and can self-renew over long periods of time, which make them promising for use in regenerative medicine [37, 38]. Due to ethical and regulatory constraints associated with ESCs, cord blood has been reported to be the most attractive source of fetal MSCs [29]. This is also because of the existence of private and public cord blood banks that makes it a convenient source for potential therapeutic applications [29]. Unlike ESCs, there has been no report of teratomas production by cord blood MSCs (CB-MSCs); thus, these cells are safer than ESCs for clinical applications [29]. In general, the umbilical cord contains 60–80 cc of cord blood (CB) that contains ESCs and MSCs, as well as hematopoietic stem cells and endothelial progenitor cells [39]. However, more studies are needed to establish the potential of CB-MSCs for tissue engineering applications.

**Figure 2:**
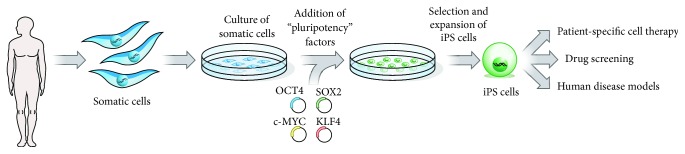
To generate iPSCs, fibroblasts (or another type of adult somatic cell) are transduced with retroviruses encoding four pluripotency factors (SOX2, KLF4, c-MYC, and OCT4). Fully reprogrammed iPSCs have similar properties to ESCs. They are competent to form teratomas on injection into mice and are capable of generating progeny. Patient's cells can be used to derive iPSCs, which can then be induced to undergo differentiation into various types of somatic cells, all with the same genetic information as the patient. Reprinted by permission from Macmillan Publishers Ltd.,* Nature*, Yamanaka & Blau [42], copyright © 2010.

It has been reported that the overexpression of a single transcription factor in somatic cells can activate cohorts of genes that are typical of other somatic cell types and can remarkably change the cell fate [40–42]. The fact that, in many differentiated somatic cell types, pluripotency can be regained through overexpression of just four transcription factor encoding genes was a breakthrough that led to the discovery of induced pluripotent stem cells (iPSCs) [42].  Figure 2 shows the steps for generating iPSCs from a typical somatic cell type (e.g., fibroblasts). Human iPSCs have been used for tissue repair and regeneration, enabling researchers to avoid the ethical and immunological issues associated with the use of embryonic stem cells (ESCs). In addition, iPSCs can be derived from a patient's own cells; therefore, they can be used to model human diseases and for drug screening* in vitro* [42]. In light of this, iPSCs are other attractive stem cell sources for tissue engineering because of their ability to be reprogramed, allowing the generation of multiple cell types from a single cell. Implantation of iPSCs in critical-sized calvarial defect of immune deficient mice has been shown to promote new bone formation and partial repair of the calvarial defect [43]. Based on recent clinical trials in Japan involving patients who had debilitating eye diseases [44], iPSCs are being considered as promising cell sources in clinical settings. However, the applications of both ESCs and iPSCs are at a preliminary stage, due to the limitations posed by ethical and political concerns, as well as the issues related to their genomic instability, immune rejection, and tumorigenesis [43].

**Figure 3:**
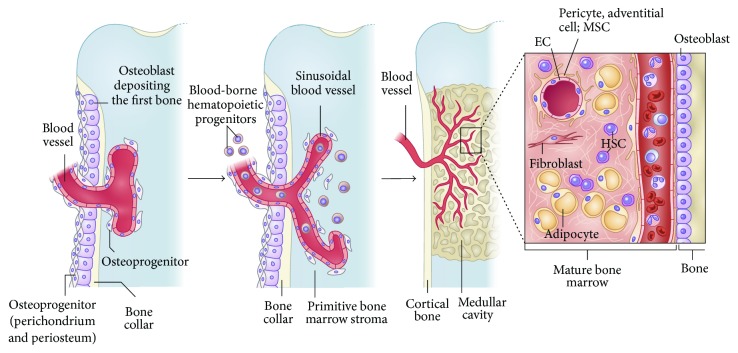
How MSCs make it to bone marrow. During development, the primitive bone marrow stroma includes skeletal progenitors that originate outside of the marrow cavity (primitive periosteum and perichondrium) and invade the forming cavity along blood vessels. Similar dynamic interactions with ingrowing blood vessels are reproduced in transplants of human MSCs and are probably the basis for the perisinusoidal position of MSCs in the intact postnatal bone marrow. Recruitment of mesenchymal cells to a mural cell fate (and a subendothelial position), a general phenomenon in development and organ growth, is mediated by endothelial cell (EC) derived PDGF-BB, which signals through PDGFR-*β* expressed on mesenchymal cells (and MSCs). Presumptive mural cells (as well as human and mouse bone marrow MSCs) produce Ang-1, which is crucial for the integrity, survival, and remodeling of vascular lattices. Ang-1 also induces quiescence of hematopoietic stem cells (HSCs). Both mural cells and endothelial cells are induced to mitotic quiescence by active TGF-*β*1, which is released through proteolytic cleavage of the latent form at sites of mural cell, endothelial cell contacts. Reprinted by permission from Macmillan Publishers Ltd.,* Nature Medicine*, Bianco et al. [46], copyright © 2013.

One of the greatest challenges in engineering of bone tissue of clinically relevant size is the mass transport limitations of nutrients and metabolic waste products [11]. Vascularization is thought to play a significant role in the healing process of tissue-engineered bone graft. The distribution of cells in a native bone tissue is usually limited to a distance of 200 *μ*m from the nearest capillary, since for oxygen and nutrients this is an effective diffusion distance [45]. The MSCs in bone marrow reside at the outer surface of sinusoids blood vessels in a subendothelial (mural) position [46].  Figure 3 shows how MSCs make it to bone marrow, as a part of a three-dimensional perivascular stromal compartment that invade the bone anlage along with the growing blood vessels. In a recent paper, Bianco et al. have elaborated on this phenomenon while reviewing the nature, identity, function, and translational aspects of MSCs [46].

**Figure 4:**
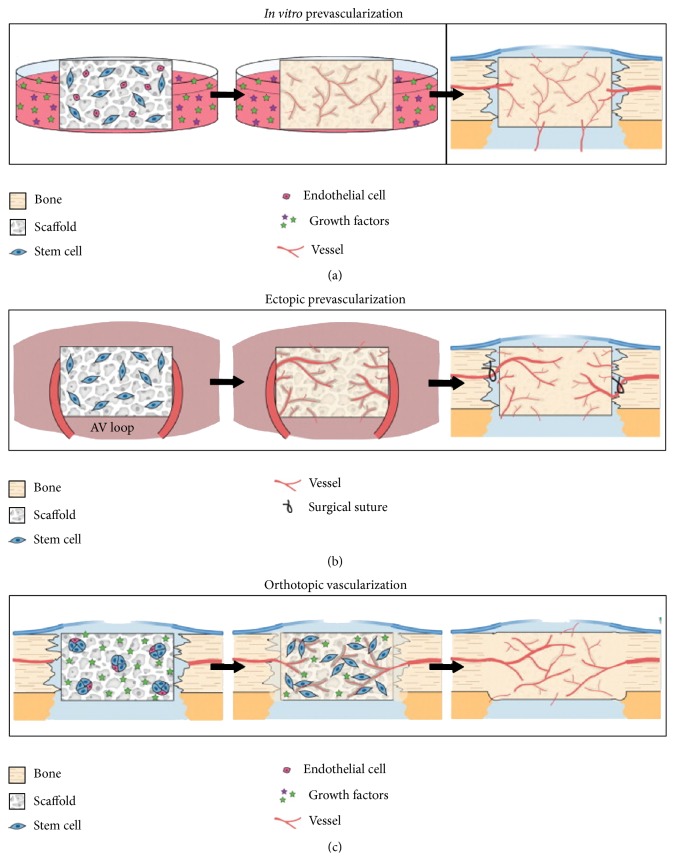
Vascularization approaches for bone tissue engineering. (a)* In vitro* prevascularization techniques induce cell-seeded scaffolds to form vasculature via exogenous growth factors. Following implantation in the bone defect, these engineered capillaries will in theory rapidly anastomose to perfuse the entire graft. (b)* In vivo* ectopic prevascularization involves implantation of a cell-seeded scaffold into a highly vascularized bed, such as muscle or arteriovenous (AV) loop, to allow extensive vascular ingrowth. The graft is transplanted as a free flap to the bone defect and surgically anastomosed with the surrounding vessels to immediately perfuse the graft. (c)* In vivo* orthotopic vascularization involves direct implantation of scaffolds into the bone defect for* in situ* tissue development. Cells seeded into the scaffolds can be aggregated to improve cell survival and endogenous cell signaling. Scaffolds can be functionalized for the controlled release of growth factors (stars) that induce bone and vascular growth. Reprinted by permission from Elsevier Ltd.,* Current Opinion in Chemical Engineering*, Hutton & Grayson [22], copyright © 2014.

The existing clinical challenges have driven efforts toward the development of strategies for vascularized bone grafts. Hutton and Grayson have reviewed several different approaches for enhancing postimplantation cell viability in bone tissue engineering, including the concept of prevascularization. Vascular networks engineered* in vitro* can serve as conduits for rapid perfusion with blood after* in vivo* implantation [22]. As shown in Figure 4, these strategies may include* in vitro* prevascularization, ectopic prevascularization, and orthotopic vascularization. These strategies have been elaborated in a recent paper by Hutton and Grayson [22].

Heterogeneous cell sources containing multiple cell types (e.g., stem cells, endothelial cells, and pericytes) have been considered by a number of research teams to promote vascularization [22]. The idea is to combine pericytes with endothelial cells (ECs), featuring an intrinsic capacity to form stable vascular structures, while the stem cells undergo osteogenesis. As a typical candidate for heterogeneous cell sources, the stromal vascular fraction (SVF) of adipose tissue contains both ASCs and ECs. Hutton and Grayson have compared various types of autologous stem/progenitor cell sources for engineering vascularized bone [22]. Robust* in vitro* culture protocols that enable synergistic development of both bone and vasculature have yet to be established. This is mainly because the biochemical cues that stimulate bone development have been shown to impede vascular growth and* vice versa* [22, 47, 48].

## 3. MSC-Seeded Scaffolds for Bone Tissue Engineering

In scaffold-based tissue engineering, the idea is to combine a porous 3D scaffold with living cells and/or biologically active molecules to form a bioengineered construct, with the ability to promote the repair and/or regeneration of biological tissues [49]. The underlying hypothesis is that the internal architecture of the scaffold can serve as a substrate to guide the formation of a structured fibrous network, which is a prerequirement for later bone formation [50]. Scaffold design for bone tissue engineering involves many parameters that affect the mechanical properties of the scaffold while directly influencing the rate of tissue regeneration [51–53]. Overcoming the current challenges in scaffold-based tissue engineering could potentially be achieved through bioinspired approaches. Designing scaffolds with nanoscale topographical features and micro-/macroscale gradient structures, combined with biological domains to interact with target growth factors, is the key for successful tissue regeneration [54]. This section reviews some of the recent studies making use of MSC-seeded 3D scaffolds for bone tissue engineering, focusing on the recommendations made in these studies to further improve cell adhesion, proliferation, and osteogenic differentiation outcomes.

To replicate the extraordinary strength and durability of natural bone, the current trend is to design biomaterials that nearly mimic the structural organization of bone from the nanoscale upward [55]. Many studies have incorporated nanostructures (such as nanoparticles or nanofibers) into scaffold formulations in order to enhance the mechanical properties of the scaffold. It has been reported that micro-/nanoscale interactions with extracellular matrix (ECM) components of the bone tissue can influence stem cell behaviors [56]. In a recent review article, Gong et al. [56] have elaborated on the classification and design of nanostructured materials, their cell interaction properties, and their application in bone tissue engineering.

**Figure 5:**
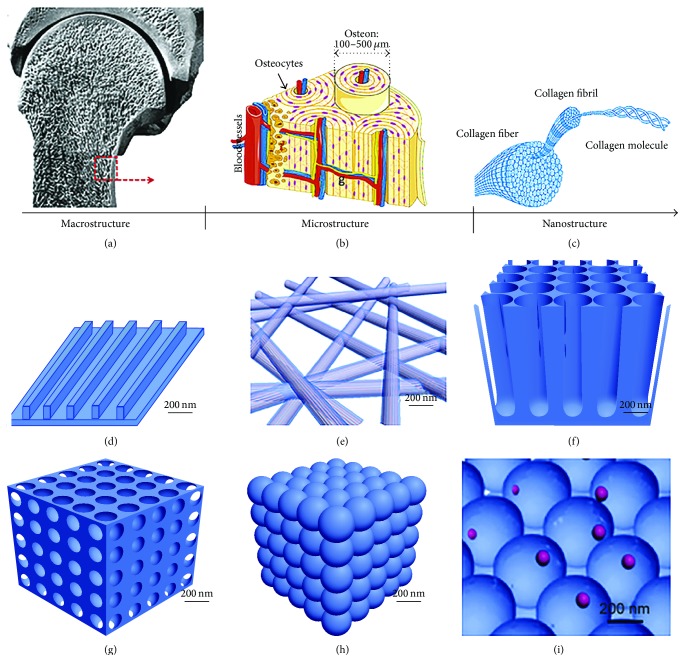
The microstructure and nanostructure of bone and the nanostructured material used in bone regeneration. (a) At the macroscopic level, bone consists of a dense shell of cortical bone with porous cancellous bone at both ends. (b) Repeating osteon units within cortical bone. In the osteons, 20–30 concentric layers of collagen fibers, called lamellae, are arranged at 90° surrounding the central canal, containing blood vessels and nerves. (c) Collagen fibers (100–2000 nm) are composed of collagen fibrils. The tertiary structure of collagen fibrils includes a 67 nm periodicity and 40 nm gaps between collagen molecules. The hydroxyapatite (HA) crystals are embedded in these gaps between collagen molecules and increase the rigidity of the bone. Nanostructures with the features of nanopattern (d), nanofibers (e), nanotubers (f), nanopores (g), nanospheres (h), and nanocomposites (i) with structural components with a feature size in the nanoscale. Reprinted by permission from Macmillan Publishers Ltd.,* Bone Research*, Gong et al. [56], copyright © 2015.

As shown in Figure 5(a), bone tissue consists of a compact shell (cortical bone) and a porous core (trabecular bone). Figure 5(b) shows the repeating osteon units within cortical bone as well as the trabeculae with bone marrow-filled free spaces within trabecular bone. These units are composed of collagen fibers (Figure 5(c)) and HA crystals, embedded within the gaps between collagen molecules to increase the rigidity of bone [56]. Given the hierarchical organization of the bone tissue, a key element in mimicking this hierarchy is to incorporate nano- and microscale features into 3D scaffolds. The commonly accepted definition of nanomaterials refers to materials with feature sizes ranging between 1 and 100 nm [56], including nanopattern [57], nanofibers [58], nanotubers [59], nanopores [60], nanospheres [61], and nanocomposites [62, 63] as depicted in Figures 5(d)–5(i) [56].

Some recent studies have combined additive manufacturing (AM) and electrospinning (ES) to produce bimodal scaffolds, so as to incorporate both nano- and microscale features into scaffold architecture [64, 65]. Additive manufacturing has been extensively used in recent years to produce prototypes of the designed scaffolds for experimental testing, enabling researchers to explore a wide range of scaffold topologies and their resulting effects on mechanical strength and tissue regeneration [53, 66–70]. On the other hand, electrospinning is a relatively simple technique to produce nonwoven mats of fibers with diameters ranging between several microns down to less than 100 nm [71, 72]. Electrospinning is considered to have unique advantages over some other scaffold fabrication techniques as it allows generating porous structures that could potentially mimic the natural ECM of biological tissues, while offering large surface areas and ease of functionalization for various biomedical applications [73, 74].

**Figure 6:**
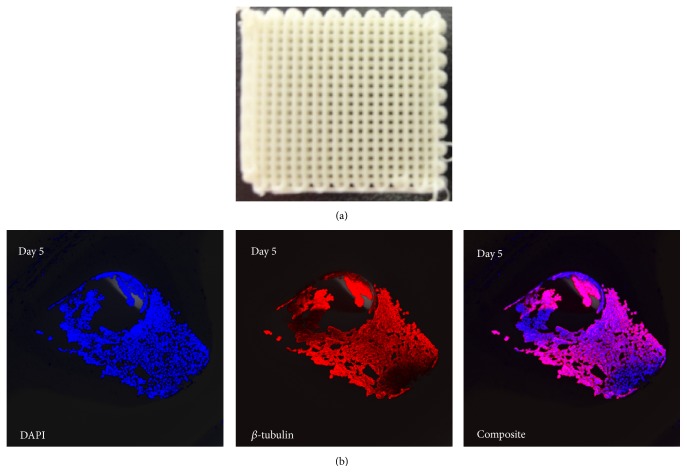
(a) Photomicrograph of a 3D-bioplotted PLGA/nHA scaffold; (b) DNA staining of the nuclei (DAPI) and anti-*β*-tubulin antibody staining of cell-seeded scaffolds on day 5 showing uniform cell adhesion onto the strands.

**Figure 7:**
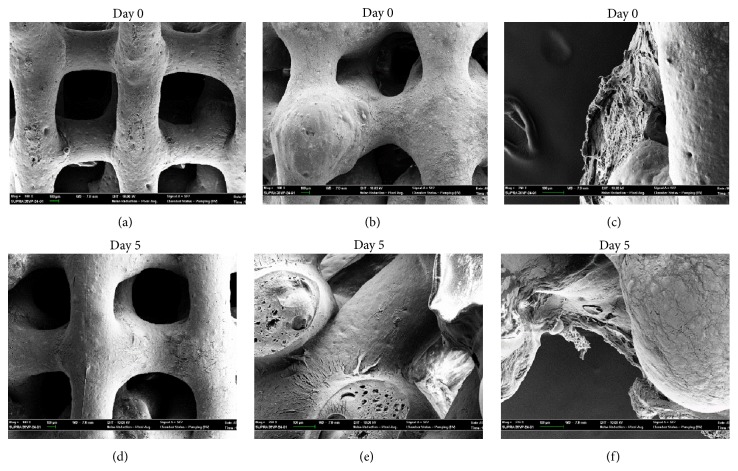
SEM micrographs of hMSC-seeded PLGA/nHA scaffolds: (a) day zero, no cells; (b, c) day zero, with cells; (d) day 5, no cells; (e, f) day 5, with cells.

Nanostructured materials with surface properties favoring cell adhesion have a greater potential for stimulating new bone growth compared to conventional materials, making them superior for tissue engineering applications [55, 75, 76].  Figure 6(a) shows a 3D scaffold made of poly(lactic-co-glycolic acid) (PLGA) and hydroxyapatite nanoparticles (nHA) produced by 3D-bioplotting technique (EnvisionTEC, Germany). DNA staining of the nuclei (DAPI) and anti-*β*-tubulin antibody staining of cells attached to these scaffolds 5 days after seeding with human embryonic kidney cells (HEK293) are shown in Figure 6(b), demonstrating a uniform cell adhesion on the strand surface. Comparing the scanning electron microscopy (SEM) images of the PLGA/nHA scaffolds before and after seeding with human MSCs reveals low cell adhesion on these scaffolds (Figure 7). This is partially due to the smooth surface of 3D-bioplotted strands, often encountered in most extrusion-based AM techniques. For scaffolds made of PLGA, a surface roughness at nanometer scale has shown to better support osteoblast functions as opposed to smooth surfaces [77]. Surface characteristics of 3D scaffolds, such as chemical composition, topography, and roughness, have been recognized as crucial factors affecting cell attachment and proliferation [78]. Micro- and nanostructured surfaces have significant effects on cell behavior [79]; therefore, combining AM techniques with conventional methods has also been explored in order to generate macro-/microporous scaffolds [80, 81].

**Figure 8:**
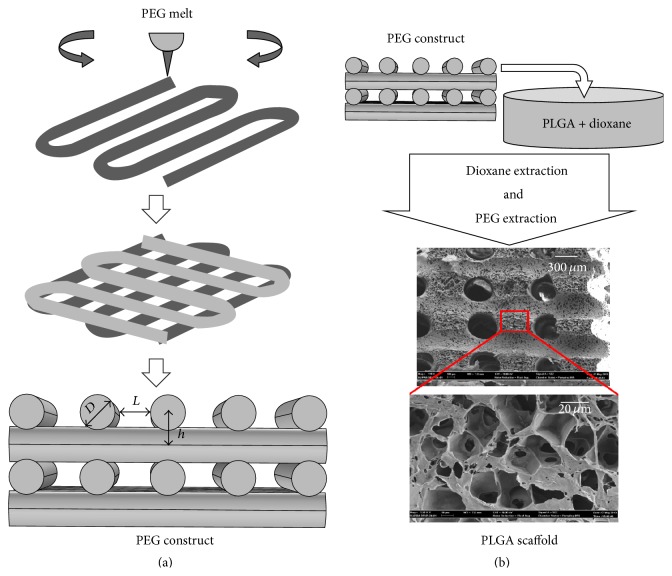
Hybrid 3D-bioplotting/TIPS scaffold fabrication technique, (a) 3D-bioplotting of the PEG constructs and the adjustable bioplotting parameters, modified from [108]; (b) schematics of the scaffold fabrication process [82].  Figure 8(a) was reprinted by permission from John Wiley and Sons:* Polymer Engineering & Science*, Yousefi et al. [108], copyright © 2007.  Figure 8(b) was reprinted with kind permission from Springer Science + Business Media:* Journal of Materials Science: Materials in Medicine*, 2015, 26:116, Akbarzadeh et al. [82], copyright © 2015.

Figure 8 shows a hierarchical scaffold produced by a hybrid 3D-bioplotting/thermally induced phase separation (TIPS) technique. While the extraction of 3D-bioplotted polyethylene glycol (PEG) can lead to interconnected macrochannels (>300 *μ*m in diameter), thermally induced phase separation produces micropores (<50 *μ*m), and nanosized surface features favorable for cell adhesion [82]. The interconnected channels played a key role in MC3T3-E1 osteoblastic cell seeding and thereby reduced the variability in cell attachment, viability, and proliferation observed in the TIPS-only scaffolds. Larger macrochannels (~490 *μ*m, compared to ~360 *μ*m) showed significantly higher cell retention, whereas the smaller macrochannels supported better cell proliferation [82]. This is consistent with curvature-driven tissue growth reported by others [83]. Surface modification of natural polymers with RGD groups containing specific molecular recognition sites has also been proposed to enhance various cellular activities on 3D scaffolds, including cell adhesion, cell-cell communication, and proliferation [84]. For example, the presence of gelatin in alginate scaffolds has been shown to enhance cell adhesion and proliferation of MSCs, while promoting the differentiation of MSCs into osteogenic cell lineage [85].

In an effort to enhance the osteogenic differentiation of MSCs within 3D scaffolds, bioceramics such as hydroxyapatite (HA) and tricalcium phosphate (TCP) have been extensively used by researchers [86–89]. Both HA and TCP have a chemistry similar to the mineral phase of natural bone. As a result, these bioceramics can promote the formation of an apatite layer on scaffold surface, leading to their integration to the host bone upon* in vivo* implantation [10, 90, 91]. Biodegradability of calcium phosphates can be controlled through regulation of the Ca/P ratio, although compounds with Ca/P ratio of less than 1 are not suitable for biological implantation due to the higher speed of hydrolysis with decreasing Ca/P ratio [92].

The nanoscale feature of hydroxyapatite nanoparticles (nHA) induces advantageous cellular responses when compared with micron-sized particles (mHA) [93]. This is mainly because the surface topography of a scaffold is one of the most crucial physical cues for cells. Both nanoscale and microscale topography can modulate cell behavior, including cell adhesion, differentiation, and proliferation [94]. Recent studies have shown that nanostructured surfaces lead to greater amounts of specific protein interactions and stimulate new bone formation more efficiently. This has led to the design of various nanostructured 3D scaffolds for tissue engineering applications [55].

Webster et al. have reported that 67 nm HA particles can significantly enhance osteoblast adhesion, when compared to conventional 179 nm HA particles after just 4 h of culture, while strikingly inhibit competitive fibroblast adhesion [16]. Nanophase ceramics have the highest adsorption of vitronectin, which is a protein promoting osteoblast adhesion. In addition, enhanced osteoclast-like cell functions and the formation of resorption pits have been reported on nHA, when compared to conventional HA [55]. Therefore, nHA is anticipated to have better bioactivity and improved biocompatibility compared to coarser crystals [95]. In another study, Cai et al. investigated the effect of nHA particles, namely, 20 ± 5, 40 ± 10 and 80 ± 12 nm in diameter, on the proliferation of BM-MSCs [96]. The* in vitro* results showed improved cytophilicity of the nanoparticles as compared with conventional HA (typically rod-like, 30–80 nm wide and 200–500 nm long). Greater proliferation of MSCs and cell viability were measured on the 20 nm sized particles [96]. Similarly, an* in vivo* study in a sheep model has shown that nHA coated metallic (Ti6Al4V) screws can provide superior bone ingrowth and osteointegration compared with mHA-coated screws [97].

Similar to calcium phosphate ceramics, bioactive glasses have many applications in bone tissue engineering due to their ability to bond to bone and promote bone growth [98]. When implanted* in vivo*, bioactive glasses induce an interfacial bioactive response. Under* in vitro* conditions, it has been reported that the ionic products from the dissolution of bioactive glasses enhance osteoblast attachment, proliferation, differentiation, and mineralization [99], while inducing the differentiation of BM-MSCs into mature extracellular producing osteoblasts [100]. It should be mentioned that bioactive glasses tend to have lower mechanical properties than cortical and cancellous bone, especially in porous form. This fact restricts the application of these materials in a wide range of biomedical applications [101].

The presence of certain ions in a doped bioactive glass can influence its biological properties [102]. For example, substituting strontium (Sr) for calcium in a bioactive glass can increase osteoblast proliferation and alkaline phosphatase (ALP) activity, while inhibiting osteoclast-mediated resorption of CaP films [103]. Since Sr has chemical and physical properties similar to calcium (Ca), it is a natural bone-seeking element and around 98% of the Sr in human body is located in bone tissues [99]. It has been documented that Sr can promote osteoblast differentiation and survival [104] and is regarded as a bone-forming agent due to its stimulation of osteoprogenitor cells replication and collagen synthesis. Nevertheless, the molecular mechanism of Sr on bone forming cells is still under investigation. A few studies have indicated the dual effects of Sr delivery by bioactive glasses: the promotion of bone formation and reduction of bone resorption [102].

## 4. Concluding Remarks

Scaffold-based bone tissue engineering using stem cells is still at its infancy. A profound scientific knowledge of each specific stem cell type is necessary to identify how to translate them to clinic, which may necessitate entirely new practices [46]. For example, tissue engineering practices that require extensive* in vitro* culture and manipulation may impose a limitation on clinical translation. Therefore, the idea of using heterogeneous cell sources (e.g., adipose and bone marrow tissues) that do not require* in vitro* culture might result in greater efficacy [22]. This is particularly important as stem cells appear to possess mechanical memory and store information from past physical environments, which can influence the cell fate [105]. Overcoming the mass transport limitations for a bioengineered bone graft will pave the way to the treatment of larger bone defects. Current tissue engineering treatments are mostly intended for relatively small defects and are immature compared to native tissue [106]. Overall, there are many hurdles on the path for the treatment of chronic degenerative diseases and in regenerative medicine using MSCs [107]. Some of the other existing challenges include guaranteeing the long-term quality of repair and avoiding potential side effects of treatment such as carcinogenesis [107].

## Figures and Tables

**Table 1:** Some stem cell sources for bone tissue engineering [22, 28, 29].

Cell Source	Advantages	Disadvantages
Bone marrow-derived mesenchymal stem cells (BM-MSCs)	(i) High osteogenic potential (ii) Studied extensively	Low abundance; requires extensive *in vitro* expansion

Adipose-derived stem cells (ASCs)	(i) Similar osteogenic characteristics as BM-MCSs (ii) Highly abundant; easy to harvest surgically	More studies are needed to test their use in bone repair

Embryonic stem cells (ESCs)	(i) Pluripotency (ii) Capable of differentiating into all cell types in bone	(i) Ethical and regulatory constraints (ii) Produce teratomas when transplanted *in vivo*

Umbilical cord blood mesenchymal stem cells (CB-MSCs)	(i) High availability (ii) Broad differentiation and proliferation potential (iii) Higher *in vivo* safety than embryonic stem cells	(i) More difficult to be isolated than MSCs from the marrow (ii) More studies are needed to test their use in bone repair

Induced pluripotent stem cells (iPSCs)	(i) Pluripotency (ii) Capable of differentiating into all cell types in bone	(i) Reprogramming efficiency is low (ii) Require extensive expansion (iii) Safety concerns; limited clinical application

Adipose-derived stromal vascular fraction (SVF)	(i) Abundant; easily harvested via liposuction (ii) Able to form vascularized bone	(i) Cell population varies among donors (ii) 2-3-hour multistep isolation process
